# Quality assurance for on‐table adaptive magnetic resonance guided radiation therapy: A software tool to complement secondary dose calculation and failure modes discovered in clinical routine

**DOI:** 10.1002/acm2.13523

**Published:** 2022-01-12

**Authors:** Carolin Rippke, Oliver Schrenk, C. Katharina Renkamp, Carolin Buchele, Juliane Hörner‐Rieber, Jürgen Debus, Markus Alber, Sebastian Klüter

**Affiliations:** ^1^ Department of Radiation Oncology Heidelberg University Hospital Heidelberg Baden‐Wurttemberg Germany; ^2^ Heidelberg Institute of Radiation Oncology (HIRO), National Center for Radiation Oncology (NCRO) Heidelberg Baden‐Wurttemberg Germany; ^3^ Medical Faculty University of Heidelberg Heidelberg Baden‐Wurttemberg Germany; ^4^ PTW‐Freiburg Freiburg Baden‐Wurttemberg Germany; ^5^ National Center for Tumor Diseases (NCT) Heidelberg Baden‐Wurttemberg Germany; ^6^ Heidelberg Ion‐Beam Therapy Center (HIT) Heidelberg Baden‐Wurttemberg Germany; ^7^ German Cancer Consortium (DKTK), Core‐center Heidelberg Heidelberg Baden‐Wurttemberg Germany; ^8^ Clinical Cooperation Unit Radiation Oncology, German Cancer Research Center (DKFZ) Heidelberg Baden‐Wurttemberg Germany

**Keywords:** adaptive radiotherapy, failure mode and effect analysis (FMEA), image‐guided radiotherapy, MR‐guided radiation therapy, online adaptive, on‐table adaptive, quality assurance, risk management

## Abstract

Online adaption of treatment plans on a magnetic resonance (MR)‐Linac enables the daily creation of new (adapted) treatment plans using current anatomical information of the patient as seen on MR images. Plan quality assurance (QA) relies on a secondary dose calculation (SDC) that is required because a pretreatment measurement is impossible during the adaptive workflow. However, failure mode and effect analysis of the adaptive planning process shows a large number of error sources, and not all of them are covered by SDC. As the complex multidisciplinary adaption process takes place under time pressure, additional software solutions for pretreatment per‐fraction QA need to be used. It is essential to double‐check SDC input to ensure a safe treatment delivery. Here, we present an automated treatment plan check tool for adaptive radiotherapy (APART) at a 0.35 T MR‐Linac. It is designed to complement the manufacturer‐provided adaptive QA tool comprising SDC. Checks performed by APART include contour analysis, electron density map examinations, and fluence modulation complexity controls. For nine of 362 adapted fractions (2.5%), irregularities regarding missing slices in target volumes and organs at risks as well as in margin expansion of target volumes have been found. This demonstrates that mistakes occur and can be detected by additional QA measures, especially contour analysis. Therefore, it is recommended to implement further QA tools additional to what the manufacturer provides to facilitate an informed decision about the quality of the treatment plan.

## 1 INTRODUCTION

The MRIdian Linac (Viewray Inc., Oakwood Village, OH, USA) provides combined magnetic resonance (MR)‐imaging and radiotherapy treatment.^1^, ^2^ Through daily acquired MR images, the initial treatment plan can be adapted to the current anatomy of the patient. During online plan adaptation, target and organs at risk (OARs) structures are recontoured and the plan is reoptimized based on electron densities derived from the initial planning computed tomography (CT).^3^ If the daily electron density map registration is imperfect or larger anatomical changes occur, the electron density map can be manually updated by structure density overwrites.

The process of daily patient imaging, image registration, contouring, replanning, and treatment delivery takes place under time pressure and is therefore prone to errors. Moreover, the adapted treatment plan cannot be dosimetrically verified by measurement before treatment, because the patient remains in treatment position during the whole procedure.^2^, ^3^ Quality assurance (QA) of online adapted treatment plans is therefore especially relevant in MR‐guided radiotherapy (MRgRT).

Several authors have reported on specific aspects of quality assurance for on‐table adaptive MRgRT. Pretreatment plan QA is often performed frequently at the time of implementation of MRgRT programs,^4^, ^5^, ^6^ and so are as well end‐to‐end tests with varying frequency.^7^, ^8^, ^9^, ^10^ During online plan adaptation, secondary dose calculation (SDC) is widely used for verification of the adapted treatment plan.^5^, ^11^, ^12^, ^13^, ^14^, ^15^

For the Viewray MRIdian Linac, the manufacturer provides an automatic adaptive quality assurance (AQA) tool that comprises a secondary dose calculation and a comparison between the initial and the adapted treatment plan.^16^ This tool recalculates doses with a secondary Monte Carlo dose calculation algorithm, using adapted structures and electron density as described above. In this way, it provides reassurance for the dose calculation process, but the result depends on its input, which is prone to a human and technical error. Suboptimal input with regard to image quality, treatment plan optimization, and contouring cannot be assessed that way.

Here, to evaluate SDC preconditions, a process failure mode and effect analysis (P‐FMEA) is warranted^17^ and was carried out at our institution prior to the implementation of MR‐guided adaptive treatments.^18^ The P‐FMEA resulted in the need for an additional plan check tool. Therefore, the automatic plan check for adaptive radiotherapy (APART) tool was developed. This work describes the possible failure modes in online adaptive radiotherapy and the way in which the APART tool can address them as well as clinical experience with adaptive treatment using the APART tool and the prevented incidents.

## 2 METHODS

The manufacturer‐provided AQA tool is mainly intended as a secondary dose calculation comprising an independent Monte Carlo dose calculation to verify that the dose of the adapted plan was calculated correctly. It also states the monitor units (MUs) of the original and adapted plan and an image‐based fluence comparison of each beam. To compare the treatment planning system (TPS) and AQA dose calculation, a gamma index pass rate is used as well as dose, dose difference, and gamma index slices through the target volume for visualization.^16^ In addition, mean doses and doses covering 1% and 99% of the volumes are provided for all contoured structures.

P‐FMEA at our institution has shown that additional automated checks of the adaptive process are necessary.^18^ A total of 84 risks were identified, whereof at least four needed the APART QA tool for risk mitigation: (i) the risk of deleting entire structures or slices within a structure, (ii) setting wrong density values (or density overwrite order) in the electron density map, (iii) mistakes in the expansion of the manually delineated gross tumor volumes (GTV) to the clinical target volumes (CTV) and the planning target volumes (PTV) and (iv) creating a too highly modulated treatment plan.

The APART tool is an inhouse‐written Matlab (The Mathworks, Inc., Natick, USA) based software tool that conducts a number of checks and analyses comparing information using exported plan information and structures sets of the original and adapted treatment plan. The output of the tool is a document with all key information for the physicist and a traffic light indicating irregularities at a first glance. To provide comprehensive information in case of doubt, it also creates a more detailed output with all analysis results of the original and the adapted plan and their structure sets.

In the clinical adaptive workflow, when the physician is satisfied with plan optimization results regarding dose coverage and OAR protection, the plan is saved and the manufacturer‐provided AQA tool is started. In parallel, the file import into the APART tool commences. The process of importing the respective files of the adapted plan, evaluating and exporting information with the APART tool, allows plan evaluation in less than 1 min. It takes place during the calculation of the AQA tool and therefore consumes no additional time. After calculation, the physicist can evaluate the results together with the results of the AQA Tool.

The APART tool assures that the correct patient plan was adapted comparing patient name and ID and treatment plan name. It compares the number of structures and calculates volume differences. If any volume deviation larger than a threshold is detected, it is stated as an alert in the output file. As the process of online adaptation and automatic contour deformation is still relatively new to our clinic, the cut‐off threshold relies on clinical experience. The precise value of thresholds was chosen from experience with the first plans as shown in the results.

In our clinic, patient‐specific margin expansions from GTV to CTV and/or PTV are chosen. For example, in a patient with several target volumes, a target that is not tracked during gated treatment will receive larger margins. These margins have to be applied daily for each fraction as target volumes are recontoured with the patient on‐table. To ensure correct margin expansions, the tool checks the consistency of volume expansions between the original and adapted plan in terms of CTV and PTV generation. It further verifies that the absolute volume changes of target structures do not differ by more than 10% and alerts if this is not the case or if there is a significant change detected, that is, if PTV volume increases while GTV decreases. It displays the resulting expansion radii supposing the target volume is a sphere for additional reassurance. It also alerts if no volume change is detected as GTV (and mostly CTV) are always recontoured in the adaptive workflow but if margin expansion is not executed, the PTV may remain unchanged.

The APART tool performs a test on the volume integrity of the structures by analyzing the DICOM RS file (which includes the patients’ structure set and ROI properties) to ensure that no unintended structure gaps occurred by accidental deletion of slices of an ROI. All applied density overrides including their priority and structures with modified electron density are depicted.

Furthermore, all segments of the double stack MLC are evaluated. The total number of segments is calculated including subsegments within one segment and is compared between the adapted and original plan. The smallest area segment with its corresponding MU as well as the smallest MU segment with its corresponding area is reported for the adapted and original plan. To enable modulation complexity calculations, the double‐stack MLC is converted into an artificial single stack MLC using the minimum united opening of both stacks.

To gain insights in the modulation complexity of the plan, various parameters are calculated such as the number of effective MUs.

$effMU_{i}=\frac{\sum_{j}\left(MU_{ij}\;*\;AA_{ij}\right)}{U(AA_{ij})}$ with *j: segment; AA_ij:_ segment area; U(AA_ij_): the union of segment areas of a beam*

for each beam *i* and also the number and size of subsegments of the plan.^19^, ^20^ An alert is sent if there are more than 10 % segments in the plan that are smaller than 1 cm^2^ or deliver less than 5 MU, as machine uncertainties have previously been quantified to be in the range of 2.4%/1.5 mm when avoiding small MLC segments with less than 5 MU.^21^

Each of the alerts mentioned has a number ascribed to it as a description of severity (see table 1). If the total number adds up to between 1 and 9, the traffic light is displayed in orange, if it adds up to more than 9, the traffic light is red.

**TABLE 1:** APART tool alerts and the assigned weight that is used to trigger the traffic light

**Alert**	**Weight**
Wrong Patient Name	+10
Wrong Plan Name	+10
MU difference larger 10%	MU Difference [%] ‐ 10
Structure was deleted	+5
Volume differences >30%	+3
Target volume differences consistent	+2
More than 10% of segments <1 cm^2^	Number of small segments [%] ‐ 10
More than 5% of segments <5 MU	+1
Gap in structure	+5

## 3 RESULTS

From February to November 2020, 62 patients were treated with adaptive radiotherapy on our MR‐Linac, most of them receiving liver (31%), lung (28%), and abdominal lymph node (28%) irradiation. During 362 of the total 466 applied fractions (77.6%) full online treatment plan adaptation including replanning was performed. In the remainder, physicians decided that it was adequate to use the original treatment plan based on clinical goals regarding target and OAR doses.

### 3.1 Viewray AQA tool

The ViewRay AQA tool was used for quality assurance in all of the evaluated 362 adapted fractions. It provided quick information on the treatment fractions and showed no irregularities in any of the fractions. SDC as stated by gamma index (2%/1 mm) agreement showed very good agreement with TPS calculation (>95%).

### 3.2 APART MU and segment analysis

**FIGURE 1:**
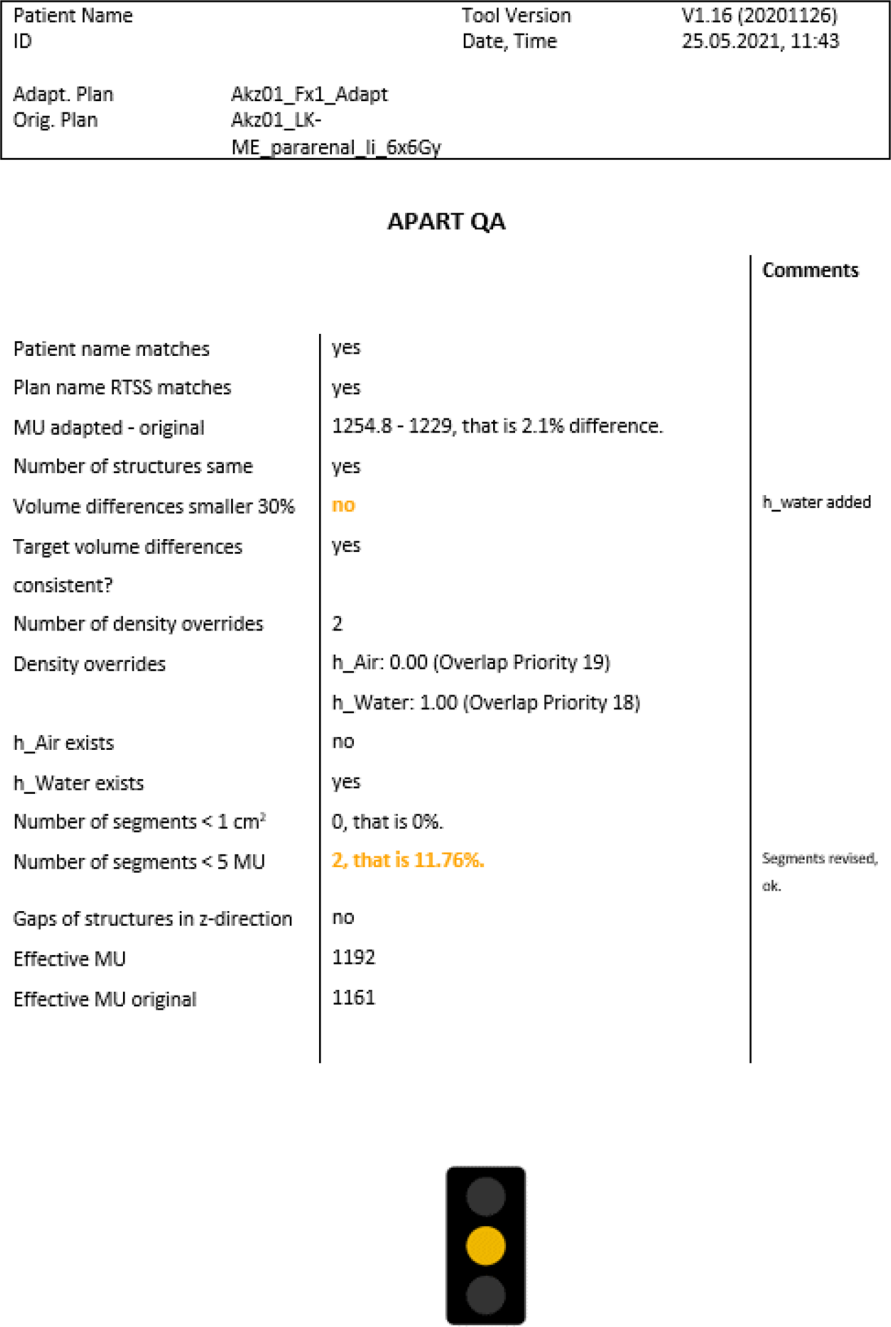
APART tool results displayed in a PDF file. Volume differences larger than 30% and two segments with less than 5 MU lead to orange traffic light

The APART tool evaluated all 362 adapted fractions and created a report as shown in Figure 1. In nine fractions, the tool detected irregularities and the adapted plan had to be changed. In a retrospective analysis, 61 acceptable adapted plans had MU differences between an original and adapted plan that were larger than 10%. One fraction with an increase in MU of 10.1% was marked untreatable due to a segment with a size of 0.66 cm^2^ and 302.6 MU. This instance was detected among 60 adapted plans with segments smaller than 1 cm^2^ or with less than 5 MU. On average, MU number decreased by −2.1 ± 19.1% through adaptation and the total number of subsegments increased by 4.6 ± 21.3%.

### 3.3 APART ROI volume analysis

**FIGURE 2:**
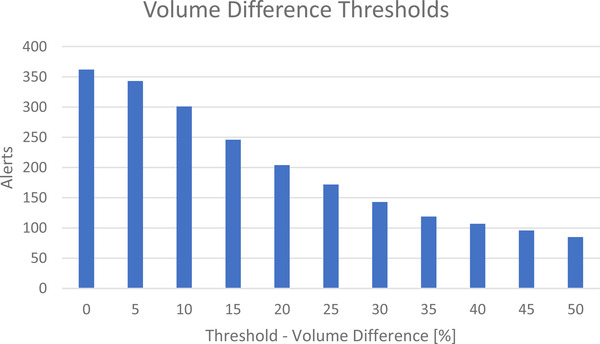
Number of adapted fractions that exhibit volume differences larger than X% between adapted contours and contours of the original plan

The tool detected no completely deleted structures in any of the adapted plans. It detected volume differences larger than 30% in 143 acceptable plans. There were no incidents related to OAR volume differences in any of the adapted plans. Figure 2 shows the number of alerts due to large volume changes for hypothetical thresholds. While there are volume changes in all instances among 362 evaluated fractions, only 85 fractions show volume changes larger than 50% in (more than) one structure.

Inconsistent target structures were detected in 126 fractions. There were four incidents related to recontouring and margin expansion of target structures: For one patient with several target volumes, the larger PTV margins for volumes that were not intended to be tracked were forgotten. This was detected through a decrease in PTV volume (−21%) while GTV and CTV volume increased (32% and 16%, respectively). This corresponds to a margin of just 1 mm for CTV to PTV uniform expansion. For one patient, the margin had been chosen too small—here PTV volume decreased by 55% (a margin of 3 mm instead of 5 mm as intended), while CTV and GTV increased by about 20%. For a patient fraction with several target structures, the wrong PTV has inflated through margin expansion accidentally. This also became visible comparing target structure volumes as to the PTV volume for the respective GTV and CTV that were edited (+19%, respectively +10%) changed by 0%. Finally, for one fraction with several target volumes, the wrong GTV was edited so that the old PTV was used for adaptation. This also became visible as the volume difference between original and adapted PTV was zero while GTV and CTV changed by −1% and −2%, respectively. None of these adapted plans were conspicuous in SDC.

PTV volumes increased by 5.9 ± 10.7% or 6.4 ± 20.3 cc on average. Intentional changes between the original and adapted treatment plan go up to 52% (down to −48%) or 209 cc intentional volume increase (−29 cc maximum volume decrease).

Among 57 adapted plans with gaps in structures, four had structures with slices that were accidentally deleted. Among those, three fractions had gaps within CTV/GTV contours. An accidentally deleted slice in the bowel of a patient became visible through the tool.

### 3.4 APART density overwrite analysis

There were no incidents related to wrong density overwrites that were used in 25 fractions (7% of all adapted fractions) to correct for imperfect electron density map registration due to air cavity changes and imperfect electron density map registration.

## 4 DISCUSSION

### 4.1 Selection of checks

Using the APART tool, risks found by the P‐FMEA could be successfully mitigated. The tool was additionally complemented by (1) treatment plan checks other authors have reported on, for example, segment size calculation,^19^ (2) checks that had already been mitigated through different measures in the FMEA but could be well implemented and documented by the APART tool, for example, comparison of MU between original and adapted treatment plan, and (3) checks for errors that appeared unexpectedly in clinical routine and had initially not been identified in FMEA, for example, non‐existing target volume changes when it was accidentally not edited. In this way, we were able to increase sensitivity for all incidents that need to be prevented during on‐table plan adaptation in the clinical routine.

Nevertheless, FMEA is never complete and objective for all cases and a recursive revision is recommended.^22^ Through continuous monitoring and improvement of our tool and regularly held risk meetings, an optimal coverage of risks through the tool and other quality assurance measures is attempted at our institution.^18^

Each of the failure modes that the APART tool covers poses distinct risks to the patient. Deleting a whole structure or structure slice might lead to wrong dose documentation in the best case and to OAR constraint violations and suboptimal target coverage in the worst case. Incorrect electron density values pose a large threat of under‐ or overdosage for the patient. Errors concerning the enlargement of target volumes also possibly lead to under‐ or overdosage of the target volume and too highly modulated fractions might exceed clinically acceptable dosimetric uncertainties.

### 4.2 Selection of action levels

The definition of precise action levels to detect critical treatment plans is challenging for adaptive treatment planning due to the high variability of patient setups, organ movement, and tumor response to treatment. For the scope of high detection sensitivity low thresholds were set for individual parameters as well as the traffic light alert. This leads to a high alert rate and causes a manual double‐check of a large number of fractions. This approach showed to be preferable compared to a plan check with high specificity. It has been shown in the presented results that fractions with inconsistencies do not necessarily show larger deviations than fractions with intended changes.

Contour volume changes have to be assessed as they can be essential for treatment outcomes. We have chosen the 30 % OAR volume difference threshold as it sends alerts in cases that require a double‐check in our opinion. This limit is subjective and is chosen so that large deviations are definitely detected. It poses a broad limit that is identical for all organs. Future versions might include patient indication‐specific margins where more critical OARs receive stricter limits than others. Larger volume changes of the structures in accepted fractions can be explained due to automatic deformation of the image, which leads to large increase or decrease in volume, especially in smaller structures. Inconsistent target structure volume changes were approved as they were mostly small changes due to partial volume effects or after manual revision of the structures and their extension. Some patients had structures with intentional gaps. On the other hand, in some cases, slices in structures were accidentally deleted during recontouring. Therefore, double checks are necessary for close cooperation with radiation oncologists.

Importantly, small segments and segments with few MUs have been monitored, which can indicate a high dosimetric uncertainty of the delivered dose. Changes in MU number can be a hint at more modulated plans but some of the increase in MU can be explained by the deeper position of PTV or the possibility to increase target coverage when organs at risk lie further apart from target structures.

The chosen thresholds within the APART tool have proven sensitive in clinical practice. The weight of the alerts was chosen so that a fraction that should not be treated necessarily appears with a red traffic light. It became clear during usage of the APART tool that the opposite is not always true; often the alerts show things that are intentional or at least unharmful for the specific patient. In this case, fractions with a red traffic light can be treated after making sure that everything is as intended. Nevertheless, APART draws attention to specific characteristics of the fraction that need to be verified or controlled by the physicist. Additionally, informed decisions are faster and easier with the detailed overview that APART provides for manual review.

### 4.3 Perspective on QA for online adaptive MRgRT

Errors that happened in the clinical routine were caught by the APART QA tool. They were invisible to SDC as it does not evaluate image contours, electron densities, and dosimetric parameters of the fraction but represents only a theoretical calculation of a “perfectly” delivered plan. Dose‐volume histograms of a planned and recalculated dose do not differ because the contour is wrong; the input to SDC is internally consistent. It is, therefore, necessary to assure the patient's treatment plan in the form of SDC input is deliverable and optimal regarding treatment objectives and dose documentation, possibly in the form of an “independent secondary contour deformation” and/or independent automatic contouring.

Also, it is necessary to implement a safety net and double‐check changes because daily decisions are made under time pressure. Much of this work can be automated with the help of software tools that assess treatment plan parameters. The APART tool has proven to be sensitive in detecting important planning mistakes and treatment objective violations. Its alerts triggering the traffic light provide a fast indication of irregularities for the physicist on duty. As it was sensitive and capable of detecting relatively small, that is, nonhazardous errors for the patient, it will prevent larger errors from happening also.

Since MRgRT has only recently been introduced into clinical practice, a consensus on QA for online adapted treatment plans has not yet been established. It is therefore important to report on outcomes of clinically applied QA procedures and detected failure modes.

The existing literature on QA for on‐table adaptive treatments is mainly focused on system‐related, technical aspects, especially pretreatment QA, SDC, and end‐to‐end tests.^4^, ^6^, ^7^, ^8^, ^9^, ^10^, ^12^, ^13^, ^15^, ^23^ Since integrated MR‐Linac systems can still be considered new technology, this is definitely warranted—but nevertheless not sufficient. Especially in the case of online adaptive treatments, only process‐based QA is able to detect specific failure modes and errors,^24^, ^25^, ^26^ and a broad discussion of applicable methods and development of appropriate tools is therefore needed.

A few authors have also reported on aspects of process QA for online adaptive MRgRT, for example, automated checks applied at each adapted fraction.^11^, ^27^, ^28^, ^29^ Compared to these, the APART QA tool was designed with a particular emphasis on the region of interest (ROI) analysis and modulation complexity. It is intended as a resource to enable quick decision‐making for the physicist approving an adapted treatment plan with the patient on‐table. For this end, it comprises simple tools such as the traffic light and the short output overview.

To our knowledge, our manuscript is the first to report in detail on failure modes that happened in clinical routine. On the other hand, single‐institution experiences can never be entirely transferred without modifications due to different circumstances. To overcome this, a larger data pool, possibly based on a collaborative incident‐reporting database specifically for on‐table adaptive treatments, could contribute to the development of detailed guidelines. Also, quality control effectiveness calculations as proposed by Ford et al.^24^ could be an effective way to evaluate and quantify different checks applied in online adaptive radiotherapy.

A limitation of the automatic evaluation of treatment plan parameters with a tool like the one presented in this manuscript is the additional data export and potential errors associated with it. The data used here for each patient fraction comprise several files that are exported from the treatment planning system and imported into the APART tool. Here, interfaces validated by the manufacturer would be helpful and should be implemented. Also, vendors should acknowledge the need for comprehensive QA tools for online adaptive radiotherapy, which become even more relevant if higher doses per fraction or even single fraction treatments are delivered as adaptive treatments. Until the manufacturer‐provided tools allow for comprehensive and configurable checks, further safety measures should be taken.

## 5 CONCLUSION

This work reports on the design, implementation, and results of a software tool for process‐based per‐fraction QA for online adaptive MRgRT. We have shown that the APART tool provides comprehensive and, in many cases, essential information for patient safety. In nine adapted fractions (2.5 %), the APART tool prevented errors that would have been undetectable with other QA measures, especially secondary dose calculation. Based on these results, the application of comparable checks is recommendable in online adaptive radiotherapy.

## AUTHOR CONTRIBUTIONS

CR performed software development, data collection, and was responsible for writing and original draft preparation. CR, OS, CKR, CB, and SK planned and designed software features. OS, CKR, CB, JHR, and SK performed patient treatment and clinical assessments. MA, SK, and JD conceived of the study and participated in its design and coordination. All the authors were responsible for data interpretation, participated in manuscript revisions, and approved the final manuscript.

## DATA VALUE STATEMENT

The data that support the findings of this study are available from the corresponding author upon reasonable request.

## CONFLICT OF INTEREST

The authors declare the following financial interests/personal relationships that may be considered as potential competing interests: SK has received speaker fees and travel reimbursement from ViewRay Inc. outside the submitted work. JHR received speaker fees and travel reimbursement from ViewRay Inc., travel reimbursement form IntraOP Medical and Elekta Instrument AB as well as a grant from IntraOP Medical outside the submitted work. JD received grants from CRI – The Clinical Research Institute GmbH, ViewRay Inc., Accuray International, Accuray Incorporated, RaySearch Laboratories AB, Vision RT limited, Astellas Pharma GmbH, Merck Serono GmbH, Astra Zeneca GmbH, Solution Akademie GmbH, Ergomed PLC Surrey Research Park, Siemens Healthcare GmbH, Quintiles GmbH, Pharmaceutical Research Associates GmbH, Boehringer Ingelheim Pharma GmbH Co, PTW‐Freiburg Dr. Pychlau GmbH, Nanobiotix A.A. as well as IntraOP Medical, all outside the submitted work.
